# The Depth of the Molecular Response in Patients with Chronic Myeloid Leukemia Correlates with Changes in Humoral Immunity

**DOI:** 10.3390/jcm13082353

**Published:** 2024-04-18

**Authors:** Michał Janowski, Karolina Łuczkowska, Michał Gniot, Krzysztof Lewandowski, Krzysztof Safranow, Grzegorz Helbig, Bogusław Machaliński, Edyta Paczkowska

**Affiliations:** 1 Department of General Pathology, Pomeranian Medical University, 70-111 Szczecin, Poland; janowskimm@gmail.com (M.J.); karolina.luczkowska@pum.edu.pl (K.Ł.); machalin@pum.edu.pl (B.M.); 2 Department of Hematology and Bone Marrow Transplantation, Poznan University of Medical Sciences, 60-569 Poznań, Poland; mgniot@ump.edu.pl (M.G.); lewandowski@ump.edu.pl (K.L.); 3 Department of Biochemistry and Medical Chemistry, Pomeranian Medical University, 70-111 Szczecin, Poland; krzysztof.safranow@pum.edu.pl; 4 Department of Hematology and Bone Marrow Transplantation, Medical University of Silesia, 40-027 Katowice, Poland; ghelbig@o2.pl

**Keywords:** chronic myeloid leukemia, cytokine, biomarker

## Abstract

**Background and Objectives**: The effective treatment of chronic myeloid leukemia leads to the restoration of proper immune system function. We aimed to investigate fluctuations in circulating cytokines, angiogenic factors and complement components in patients with CML during the first year of treatment with TKI and correlate them with the degree of achieved molecular response. **Material and Methods**: We recruited 31 patients with newly diagnosed CML. Peripheral blood and bone marrow samples were obtained, and concentrations of serum proteins were measured using an immunology multiplex assay. **Results**: The study cohort was divided into two groups of optimal or non-optimal in accordance with the European Leukemia Net (ELN) guidelines. We found significantly higher concentrations of C1q, C4 and C5a in serum after 3 months of TKI treatment in patients who achieved optimal responses in the 6 months after diagnosis. The most alterations were observed during 12 months of therapy. Patients in the optimal response group were characterized by higher serum concentrations of TGF-β, EGF, VEGF, Angiopoietin 1, IFN-γ and IL-8. **Conclusions**: The later plasma concentrations of complement components were significantly increased in patients with optimal responses. The changes after 12 months of treatment were particularly significant. Similar changes in bone marrow samples were observed.

## 1. Introduction

Chronic myeloid leukemia (CML) is a type of neoplasm characterized by reciprocal translocation between the long (q) arms of chromosomes 9 and 22 [1]. This crucial genetic event is a reason for the creation of the pathological BCR/ABL fusion gene [2]. In turn, the aforementioned oncogene is a template for the protein constitutively active tyrosine kinase, which is a paradigm for CML. This cytogenetic foundation represents a target for drugs named tyrosine kinase inhibitors (TKI), which include imatinib, nilotinib, dasatinib, bosutinib and the novel drugs ponatinib and asciminib. This pragmatic approach to the treatment of CML revolutionized hematology and profoundly changed prognosis and overall survival in CML. The current trend in clinical practice is even more brave and assumes the induction of a prolonged and profound molecular response in the course of treatment as a prelude to the successful cessation of therapy with TKIs [3]. This phenomenon is named treatment-free remission (TFR), and according to the current guidelines, it is a goal of treatment in some patients. However, it is clear that this “functional treatment” cannot be explained solely by oncokinase inhibition. It can be concluded that the restoration of the immune system during TKI treatment is even more important, especially in maintaining remission. In this respect, the renewal of the immune system consists of the restoration of balance between the effector and immunosuppressive roles [4]. Unfavorable treatment scenarios, including resistant disease, progression to the blastic phase and the unsatisfactory depth of molecular response to the treatment, have prompted the search for responsible factors. Immune pathways seem to be crucial in understanding the individual response to TKIs. It has not been fully elucidated which patients are candidates for the discontinuation of treatment. The rate of successful TFR events ranges in most clinical trials from 41% (STIM) to 67.9% (STAT2), and a large proportion of patients experience the recurrence of CML [5]. Thus, new factors responsible for the efficacy of the cessation of treatment are in demand. The current state of knowledge regarding immunological control during TKI treatment indicates significant changes in the cell-mediated immune response. For example, the increased number of more mature NK cells, cytotoxic T lymphocytes, has been well proven. Simultaneously, patients with deep molecular responses are characterized by a reduced number of suppressor cells such as myeloid-derived suppressor cells (MDSCs) and regulatory T cells (Tregs) [6]. Less is known about the changes that occur in plasma proteomics in patients undergoing TKI therapy. There are studies investigating the variability in plasma cytokine profiles during TKI therapy. Imatinib affects monocyte chemotactic protein 1 (MCP-1) and osteoprotegerin (OPG), the levels of which are significantly lower during TKI treatment in comparison to healthy donors [7]. During TKI treatment, a variation in complement levels was observed [8]. The analysis of proteomics differential expression profiles revealed significant differences among patients according to the depths of molecular response [9]. Given potential of the Luminex method, which allows the simultaneous measurement of many factors in the same sample, we aimed to study the plasma and bone marrow concentrations of 40 different substances in consecutive time points at diagnosis and during TKI treatment. The following plasma proteomics were analyzed in our study: immunoglobulins (IgM, IgG1, IgG2, IgG3, IgG4, IgA), complement proteins (C1q, C3, C3b, C4, FB, FH, FI, C2, C4B, C5, C5A), interleukins (IL-1, IL2, IL4, IL-6, IL-8), interferon gamma (IFN-γ), tumor necrosis factor-α (TNF-α), tumor necrosis factor-β (TNF-β), growth factors (platelet-derived growth factor—PDGF; epidermal growth factor—EGF; vascular endothelial growth factor—VEGF; fibroblast growth factor—FGF; basic fibroblast growth factor—FGFb; fibroblast growth factor—acidic—FGFA; placental growth factor—PlGF; hepatocyte growth factor—HGF; transforming growth factor beta 1—TGFB1), hematopoietic growth factors (granulocyte colony-stimulating factor, G-CSF; granulocyte-macrophage colony-stimulating factor, GM-CSF), matrix metallopeptidase (MMP), C-C motif chemokine ligand 5 (CCL5), tissue inhibitor of metalloproteinase-1 (TIMP-1) Angiopoietin-1, Stress-Associated Endoplasmic Reticulum Protein 1 (SERP1), leptin, thrombospondin-2 and endostatin.

## 2. Materials and Methods

### 2.1. Patients

The study was conducted in three hematological centers in Poland: the Department of Hematology and Bone Marrow Transplantation, Pomeranian Medical University, Szczecin, Poland; the Department of Hematology and Bone Marrow Transplantation, Poznan University of Medical Sciences; and the Department of Hematology and Bone Marrow Transplantation, Medical University of Silesia, Katowice. Prior to enrolment, all patients signed informed consent in accordance with the Declaration of Helsinki. Before patient enrolment, we obtained appropriate approval from the local ethics committee of the Pomeranian Medical University (approval code: KB-0012/11/2021). All patients had previously undergone routine hematological diagnostics aimed at establishing the diagnosis. All patients were diagnosed with chronic-phase (CP) CML, with a transcript type that can be monitored using the real-time PCR method according to the ELN guidelines. None of the patients had undergone prior treatment before introducing TKI. To assess the effect of TKI treatment on the serum and bone marrow proteomics and cytokines of innate immune response, we collected serum plasma samples at four consecutive time points. Additionally, we collected bone marrow samples at the same subsequent time points. Plasma samples from CP-CML patients enrolled in our trial were collected at the baseline (*n* = 12); after 3 months on TKI (*n* = 13); after 6 months on TKI (*n* = 15); and after 12 months on TKI (*n* = 13). We divided the study cohort into two groups according to the obtained response, optimal (OPT) and non-optimal response (NOPT), in accordance with the European Leukemia Net (ELN) guidelines. The criteria for either classification outcome in the OPT group vs. NOPT group was the achievement of the following milestones: at 3 months—level of BCR-ABL^IS^ ≤ 10%; at 6 months—level of BCR-ABL^IS^ ≤ 1%; at 12 months—level of BCR-ABL^IS^ ≤ 0.1%. Patients were considered to have a non-optimal response (NOPT) if BCR-ABL1 transcript reduction could not be demonstrated below the level assumed above. The study cohort is summarized in Table 1; the inclusion and exclusion criteria for the study are summarized in Table 2.

### 2.2. Multiplex Assay

A specimen of the peripheral blood (5 mL) was obtained from all participants, and the plasma samples were separated. We used complex panels containing 40 biomarkers, among others: immunoglobulins (IgM, IgG1, IgG2, IgG3, IgG4, IgA) interleukins (IL-2, IL-4, IL-6, IL-8), complement proteins (C1q, C3, C3b, C4, factor B, factors H, C2, C4B, C5, C5A), growth factors (EGF, VEGF, G-CSF, GM-CSF, PDGF), cancer-related proteins (IFNy, TGF-β) and others. Concentrations of plasma proteins were measured in peripheral blood plasma by the Luminex method based on color-coded superparamagnetic beads coated with analyte-specific antibodies (Luminex Corporation, Austin, TX, USA) using the commercial Luminex Human Discovery Assay (3-plex) kit (R&D Systems, Minneapolis, MN, USA). The procedure was performed according to the manufacturer’s protocol. In short, 50 µL of blank standards and samples was added to a 96-well plate and incubated with a microparticle cocktail for 2 h in the dark, at room temperature (RT), on a horizontal–orbital microplate shaker set at 750 rpm. After the incubation time elapsed, the wells were washed three times with 1 µ of wash buffer (100 µL/well). In the next step of the procedure, 50 µL of the biotin–antibody cocktail was added to the plate and incubated for 1 h in the dark, at RT, on the horizontal–orbital microplate shaker (750 rpm). In the last step of the procedure, streptavidin-PE (50 µL/well) was added to the plate and incubated for 30 min under the same conditions as the previous steps. Finally, the microspheres on the plate were washed three times, resuspended in wash buffer (100 µL/well) and read on the Luminex 200 analyzer (Luminex Corporation, Austin, TX, USA).

### 2.3. Statistical Analysis

Non-parametric tests were applied for quantitative variables because their distributions significantly differed from the normal distribution. The Mann–Whitney test was used to compare values between the OPT and NOPT group and the Wilcoxon signed-rank test was used to compare values obtained at different time points. We considered *p* < 0.05 statistically significant. Statistica 13 software (Dell Inc., Oklahoma City, OK, USA) was used for statistical analysis. The R 4.3.2 environment and the ggplot2 package were used for the graphical presentation of data.

## 3. Results

### 3.1. Patients Characteristics

A total of 31 patients (16 males, 15 females) aged 33 to 74 years (median 50.5; mean 52.4 years) were included. All patients underwent treatment with tyrosine kinase inhibitor—initially imatinib. Eight patients received second-line therapy (three nilotinib and five dasatinib), all because of therapy failure. The risk classification in Sokal score was as follows: 61% low risk, 33% intermediate risk and 6% high risk. A total of 3% of patients were classified as high risk on the EUTOS scale. The initial parameters of peripheral blood count and IS% values during treatment are presented in Table 3.

### 3.2. Significantly Changed Proteomics Levels Depended on Response

**Figure 1:**
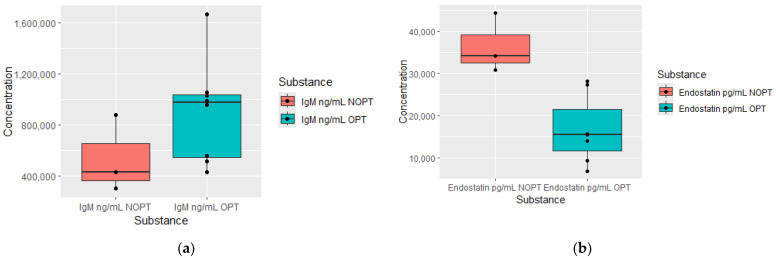
Plasma levels of proteomics measured at diagnosis in patients who achieved optimal response (OPT) at 3 months of treatment and plasma levels in non-optimal response group (NOPT). (**a**) Comparison of IgM concentrations (OPT *n* = 8, NOPT *n* = 3); (**b**) comparison of endostatin concentrations (OPT *n* = 7, NOPT *n* = 3).

At diagnosis, patients in the OPT group after 3 months of treatment had significantly higher IgM levels and decreased endostatin concentrations. The optimal response at this time point was also equal to an early molecular response. This data is presented in Figure 1.

**Figure 2:**
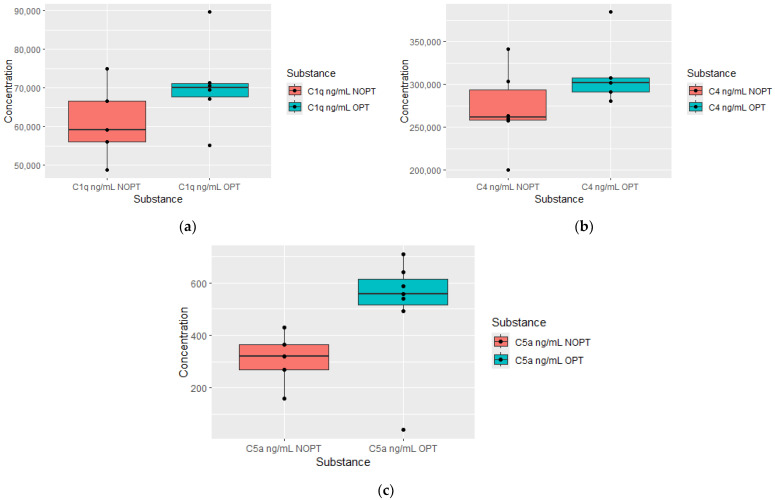
Plasma levels of proteomics measured after 3 months of treatment in patients who achieved optimal response (OPTs) after 6 month of treatment and plasma levels in non-optimal response group (NOPT). (**a**) Comparison of C1q concentrations (OPT *n* = 5, NOPT *n* = 5); (**b**) comparison of C4 concentrations (OPT *n* = 5, NOPT *n* = 6); (**c**) comparison of C5a concentrations (OPT *n* = 7, NOPT *n* = 5).

For the purpose of indicating potential plasma proteomics which could act as predictors of better response, we investigated which substances were altered at previous time points (3 and 6 months) in the OPT group 6 and 12 months after diagnosis. We found significantly higher concentrations of C1q, C4 and C5a in plasma after 3 months of treatment in patients who achieved optimal responses in the 6 months after diagnosis compared to the non-OPT group. This data is presented in Figure 2.

**Figure 3:**
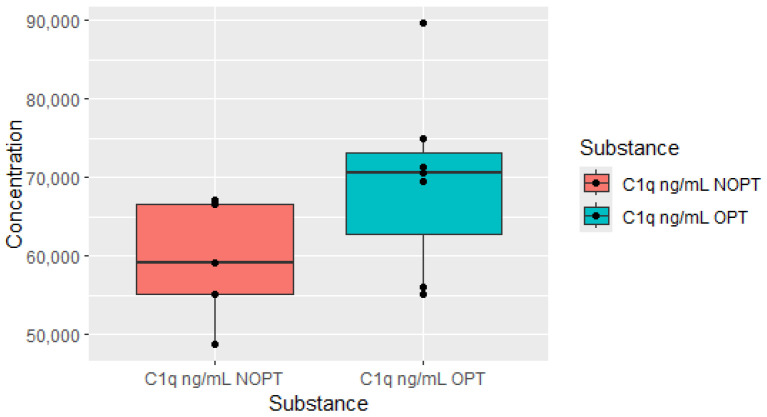
Plasma levels of C1q measured after 3 months of treatment in patients who achieved optimal response (OPTs, *n* = 7) after 12 months of treatment and plasma levels in non-optimal response group (NOPT, *n* = 5).

As well as in the six-month group, C1q was also elevated 3 months after diagnosis in the OPT group at 12 months from baseline, which is shown in the Figure 3.

**Figure 4:**
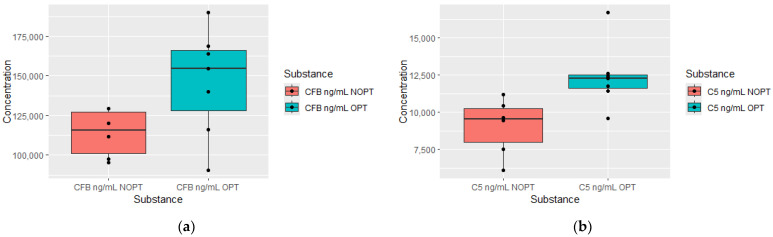
Plasma levels of Complement Factor B (CFB) measured after 6 months of treatment in patients who achieved optimal response (OPTs, *n* = 7) and non-optimal response group (NOPT, *n* = 5) after 12 months of treatment (**a**) and plasma levels of C5 measured after 6 months of treatment in patients who achieved optimal responses (OPTs, *n* = 7) and non-optimal response group (NOPT, *n* = 6) after 12 months of treatment (**b**).

Patients in the OPT group at 12 months had higher plasma Complement Factor B (CFB) and C5 concentrations 6 months after diagnosis (Figure 4).

**Figure 5:**
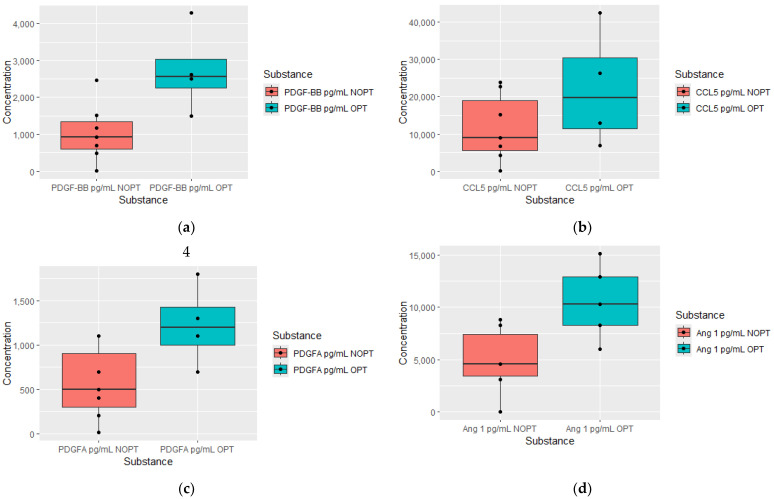

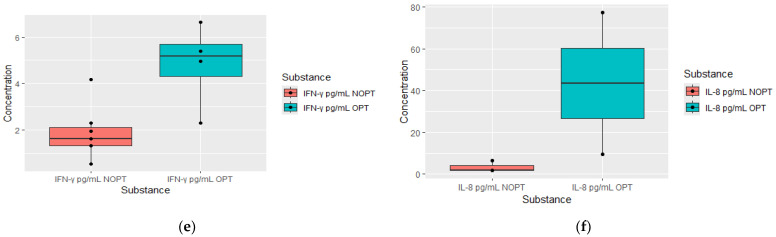
Plasma levels of proteomics measured after 12 months of treatment in optimal response group (OPT), and non-optimal response group (NOPT). Platelet-derived growth factor—BB (PDGF-BB) OPT *n* = 4, NOPT *n* = 7 (**a**), C-C motif chemokine ligand 5 (CCL5) OPT *n* = 4, NOPT *n* = 7 (**b**), platelet-derived growth factor subunit A (PDGFA) OPT *n* = 4, NOPT *n* = 6 (**c**), Angiopoietin 1 OPT *n* = 5, NOPT *n* = 5 (**d**), interferon gamma (IFN-γ) OPT *n* = 4, NOPT *n* = 6 (**e**), IL-8 OPT *n* = 3, NOPT *n* = 7 (**f**).

We observed the most alterations in plasma proteomics 12 months after TKI therapy initiation (Figure 5). Patients in the OPT group were characterized by higher plasma concentrations of substances such as platelet-derived growth factor—BB (PDGF-BB), C-C motif chemokine ligand 5 (CCL5), platelet-derived growth factor subunit A (PDGFA), Angiopoietin 1, interferon gamma (IFN-γ) and IL-8. In Table 4, we summarize all proteomics measured at individual measurement moments.

In order to verify relevant findings in the profiles of serum proteins, we conducted the same assessment in bone marrow samples. Only at the 12 month time point in the observation did we find statistically significant changes in the bone marrow. Bone marrow obtained from the OPT group had higher concentrations of TNF-α, IL-8, VEGF, basic fibroblast growth factor (FGFb) and fibroblast growth factor—acidic (FGDA). These findings are summarized in Table 5.

We analyzed the variability in plasma proteins between the designated time points in all studied patients, independently of the degree of response. We noticed significant reductions in serum TNF-α, VEGF, matrix metalloproteinase-8 (MMP-8), thrombospondin-2 and C5 and C2 complement proteins during first 3 months of treatment; conversely, the concentration of GM-CSF increased in the same period. When comparing the values between 3 and 6 months of treatment, reductions in levels of Agniopoetin-1 and IgG2 were observed. In this comparison, the serum IgM concentration accumulated. The endostatin and C2 concentrations slightly increased in the last time period between 6 and 12 months. These data in relation to statistically significant changes and immune-relevant serum proteins are shown in Table 6.

## 4. Discussion

In our work, we aimed to investigate the relationship between the depth of the molecular response and alterations in plasma and bone marrow proteins. For this purpose, we correlated protein concentrations with the depth of response at the baseline and after 3, 6 and 12 months of TKI treatment. The next step was to divide the patients into two groups, depending on the clinical response. The use of a broad panel of studied proteins, including cytokines, chemokines, and growth factors, allowed us to obtain significant insight into changes occurring in the serum and bone marrow of a CML patient.

We observed that a decreased endostatin concentration at the baseline was linked with an early molecular response in the 3rd month. Endostatin, which acts as an angiogenesis inhibitor, is a promising object of research in the treatment of solid tumors [10]. The importance of endostatin in CML has not been elucidated. Contradictory reports are available with regard to the matter of significance of endostatin in myeloid neoplasms. In one study, elevated serum endostatin levels were associated with favorable outcomes in acute myeloid leukemia (AML) [11]. In turn, another study indicated a relationship between elevated serum endostatin levels and shorter survival in patients with myelodysplastic syndrome (MDS) and AML [12].

Among the investigated immunoglobulins, the only one elevated was IgM, observed at diagnosis in patients in the OPT group in the 3rd month of treatment. It has been previously reported that CML treatment responders showed an increased IgM concentration in bone marrow and peripheral blood plasma samples. Moreover, these IgM were reactive with leukemic cells, leading to the death of themselves in a complement-independent way [13]. On the other hand, it has been shown that imatinib treatment decreased plasma IgA and IgG levels, while dasatinib reduced IgM levels [14].

An important finding of our work is the observation of the increased serum concentration of complement components in patients who achieved an optimal response to treatment (OPT group). Moreover, elevated components of the complement cascade always preceded the achievement of better therapeutic effects in the study cohort. Thus, higher C1q, C4 and C5a concentrations in the 3rd month occurred in patients in the OPT group in the 6th month, and higher Complement Factor B (CFB) and C5 concentrations at 6 months heralded a better response at 12 months. Independently, patients in the OPT group at 12 months had higher C1q concentrations after 3 months of treatment. Complement components, as part of the innate immune response, play pivotal roles in anti-microbial mechanisms but are also present in the tumor microenvironment. In addition to the well-known and expected role in anti-tumor activity of complement components, there is strong evidence that complement cascades also exhibit pro-tumoral actions. Research in recent years has indicated that the diversity of biological effects of complement components depends on the type of cancer and often has opposite effects [15]. So far, the role assigned to complement components in CML patients has not been clearly explained. The diversity of actions allows us to distinguish different complement phenotypes. Thus, cancers are distinguished by complement components having protective, aggressive and uncertain significance [16]. The complement component 1q (C1q), together with C1r and C1s, forms the C1 complex and activates the classical pathway of the complement cascade. This molecule has various biological functions. Some of these non-canonical functions are fields of interest of oncology [17]. The plasma level of C1q is positively correlated with the remission status of multiple myeloma [18]. On the other hand, in acute myeloid leukemia, which is closely related to CML, C1q-positive leukemic cell subsets have been shown to act as markers of adverse prognosis in AML and early recurrence, and finally, C1q-positive leukemia cells have shown the ability to infiltrate tissue [19]. An important difference is the methodology of the aforementioned study, which used single-cell RNA sequencing to identify gene expression. To date, the significance of C1q particles in the course of CML remains unknown. However, data from our experiment support the thesis of the beneficial role of C1q. We noticed elevated levels of C4 in patients after 3 months of TKI treatment, who were in the OPT group in the 6th month. In a study by Humlova et al., increased levels of C4 in pretreated patients dropped in the course of pharmacotherapy [8]. We found significantly increased C5a levels measured at 3 months, and similarly, an increase C5 was measured at 6 months, which were both in the OPT group in consecutive months (6 and 12 month). In CLL, abnormal patterns of C5 were observed, conversely to the levels of C3 and C4, which were similar in a population of CLL patients [20]. CFB was normalized after the treatment of acute lymphoblastic leukemia [21]. In our experiment, patients in the OPT group, after twelve months of treatment, had increased CFB levels from six months earlier. However, the CFB concentrations in both groups (OPT vs. NOPT) did not differ in the twelfth month. It is worth emphasizing the observation regarding the increased concentration of components responsible for both the activation and inhibition of complement components. In our study, increased levels of C5, C4 and inhibitory factor B occurred in the preceding time points in patients with optimal responses to treatment.

The twelfth month of treatment brought the greatest variability in plasma protein concentrations measured in the OPT group. Particularly controversial is the increased concentration of various growth factors, such as PDGF, EGF, VEGF and Angiopoietin-1, whose role in the pathogenesis of hematological cancers is clearly negative. The elevated levels of VEGF may influence the outcome in a negative way by causing leukemic cell growth and promoting angiogenesis [22]. On the contrary, in our study, VEGF and EGF levels systematically decreased at subsequent time points in the entire study group (statistically insignificant results), which is consistent with the current state of knowledge.

IL-8, a chemoattractant cytokine, is involved in the pathogenesis of numerous types of cancers. It seems to play a clearly negative role in progression, e.g., in the transition of tumor cells to a mesenchymal phenotype and the development of metastases. The blockade of IL-8 receptors is an area of research in various solid tumors, including breast cancer [23]. It is well proven in the field of myeloid tumors that IL-8 is overexpressed in AML, MDS and primary myelofibrosis patients (PMF) [24]. In our clinical experiment, IL-8 was significantly higher in patients who reached the 12-month treatment milestone. Similar results, which seem to confirm ours, were obtained in another study evaluating plasma proteomics in CML patients [25]. In this study, IL8 levels were significantly higher before treatment in patients responding well to treatment, and the result was confirmed in the following months. A study by Petrackova et al. supports these observations of positive correlations between plasma IL-8 levels and optimal responses in the course of CML treatment [26]. Our observations are in contrast to the study by Hantschel et al. [27], who demonstrated in cell and clinical models that the gene expression of IL-8 is downregulated by dasatinib, and the plasma levels of IL-8 decreased to baseline during imatinib treatment. It is worth emphasizing that in the cited experiment, another TKI (dasatinib) was used in the cell culture, and plasma levels were assessed in four patients, of which, in one patient, an increasing level of IL-8 was found despite reaching a complete cytogenetic response. The above data do not determine the prognostic significance of IL-8. Perhaps its role in CML is different from that observed in solid tumors and AML/MDS. In our cohort, the plasma levels of IL-8 did not differ significantly over particular time points.

We found an elevation in TGF-β in patients with a good response at 12 months of treatment in our cohort. For instance, transforming growth factor α (TGFα) is a growth factor which is expressed in myeloid blast origin from CML cells [28]. TGFα exerts cancer-promoting effects in many ways. In the context of cancer immunology, it is important to mention helping malignant cells in immune system evasion. TGFα has been identified as a factor determining worse survival in patients with AML and acute lymphoblastic leukemia (ALL) [29]. In CML, Nievergall et al. showed that high TGF-α and IL-6 levels strongly predict failure to achieve early molecular response [30]. Conversely, TGF-β can act as an effective growth inhibitor and promote cell apoptosis, thus inhibiting the occurrence and development of tumors [31]. Some studies suggest that disturbance in TGF-β signaling in CML facilitates entry into blast crisis [32]. In a large genetic study, TGFB1 expression in CML was positively correlated with levels of NK and T, which revealed a tumor-suppressive role [33]. There is concordance between our findings and the aforementioned studies. The TGF-β signaling pathway appears to play an important role in the recovery of immune surveillance in CML; thus, the elevation of TGFB in patients with optimal responses at 12 months of treatment may be explained by this.

We are very cautious about the CCL5 elevation in the OPT group in the 12th month, because the CCL5/CCR5 axis is suspected to facilitate tumor progression [34]. This finding certainly requires more detailed investigation in a larger group of patients.

In recent years, the idea of using interferon-α with TKI has been experiencing a renaissance [35,36]. Only in the 12th month of observation were the OPT-group patients characterized by higher INF concentrations. This observation is consistent with other reports [26].

### Limitations

The findings of this study have to be considered in light of some limitations. First, this analysis, despite the multicenter nature of the study, was conducted among a small-size cohort. This is the reason why our results need to be confirmed in longitudinal studies. Finally, we only analyzed patients’ plasma. We realize that analyzing serum and plasma separately would provide more information, as many proteins are degraded in plasma while they are stable in serum.

## 5. Conclusions

In conclusion, the results of our study shed light on the relationship between the depth of molecular response, expressed as achieving milestones, and circulating plasma proteins. Despite slight variability in concentrations among all study members at subsequent time points, their stratification according to molecular response allowed the identification of a number of substances whose measurement may be useful. Our results fit into the overall research on plasma proteomics in CML. This study may provide a new perspective on the possible prognostic factors of achieving optimal responses in CML patients. We believe that future research should proceed in the field of plasma protein alterations during TKI treatment, not only in CML.

## Figures and Tables

**Table 1:** Patients’ characteristics (*n* = 31).

Age at Diagnosis (Years)	50.5 (33–74)
Sex (N (%))	
Female	15 (51%)
Male	16 (49%)
Sokal score	
Low risk	19 (61%)
Intermediate risk	10 (33%)
High risk	2 (6%)
EUTOS score (N (%))	
Low risk	28 (90%)
High risk (7)	3 (10%)
TKI	
Imatinib	23 (74%)
Dasatinib	5 (16%)
Nilotinib	3 (10%)

**Table 2:** Inclusion and exclusion criteria for participation in the study.

Inclusion Criteria	Exclusion Criteria
Age over 18 and under 80	Diagnosis of chronic myeloid leukemia in the accelerated or blast phase
Diagnosed with chronic myeloid leukemia in the chronic phase	Coexistence of another myeloid or lymphoid tumor
No prior TKI treatment for CML	Reservations from a clinician that they would qualify to participate in the study regarding compliance with medical recommendations

**Table 3:** Clinical characteristics of the patients. Peripheral blood morphology parameters at the time of diagnosis and IS% values in the following months.

	Morphology at Diagnosis			IS%		
No.	WBC × 10^9^/L	Hb(g/L)	PLT × 10^9^/L	3rd Month	6th Month	12th Month
1	147.06	11.5	171	12.84	1.6442	0.74
2	222.54	5.4	136	87.09	17.53	10.48
3	165	10.1	270	0.54	0.07	0.01
4	113	11.2	173	8.92	0.56	0.41
5	208.55	11.4	368	23.26	19.37	0.0074
6	39.45	13.8	210	2.27	0.3	2.14
7	32.89	10.8	1134	7.15	0.022	0.038
8	45.77	12.1	627	5.63	4.95	0.179
9	36	12.8	212	12.3	8.5	0.38
10	134	13	340	3.4	0.12	0.8
11	487	11.1	396	10.6	6.9	0.27
12	216	8.6	1484	34.89	9.2	0.13
13	57.94	12.7	256	2.62	1.27	1.24
14	224.18	9.4	807	9.35	0.13	0.007
15	47.78	12.4	840	3.57	3.99	0.06
16	151.85	11.6	131	1.36	0.838	0.04
17	86.2	12.9	1060	14.84	8.47	103.69
18	59.05	12.3	522	6.66	0.24	0.23
19	449.73	10.8	652	142.30	2.098	0.0085
20	392.84	6.1	334	9.98	0.027	93.42
21	127.82	10.2	287	4.86	3.25	10.95
22	82.62	14	378	21.73	16.06	2.12
23	317.89	13.3	141	131.02	0.003	0.04
24	137.2	9	623	0.17	6.39	1.38
25	15.03	14.17	573	9.03	0.12	0.056
26	86.98	14	188	1.47	2.44	0.13
27	46.02	12.4	666	9.59	0.38	0.07
28	85.44	10.8	768	7.71	0.13	3.99
29	430.61	11.3	306	9.35	0.13	0.38
30	47.78	12.4	840	1.19	0.17	0.13
31	27.76	13.8	230	2.51	29.23	0.13

**Table 4:** Levels of cytokines in plasma samples. Median concentrations and ranges are listed. *p*-values were derived from Mann–Whitney tests.

	Optimal Response (OPT)	Non-Optimal Response (NOPT)	*p*-Value	*n* (OPT and NOPT)
Serum cytokine levels measured at diagnosis in patients who achieved early molecular response in 3 months				
Endostatin µg/mL	15,7 (6.9–28.1)	37.5 (30.7–44.3)	0.0299	OPT = 7, NOPT = 3
IgM ug/mL	1029.8 (432.6–1668.2)	365.1 (299.51–430.7)	0.0299	OPT = 8, NOPT = 3
Serum cytokine levels were measured at 3 months, depending on response according to ELN after 6 months of treatment				
C1q µg/mL	69.6 (55.8–89.7)	56.1 (48.6–66.6)	0.0136	OPT = 6, NOPT = 5
C4 µg/mL	307.6 (280.4–384.7)	260.3 (199.5–303.4)	0.0136	OPT = 5, NOPT = 6
C5a µg/mL	539.8 (39.5–709.1)	320.2 (158.4–430.8)	0.0327	OPT = 7, NOPT = 5
Serum cytokine levels measured at 3 months, depending on response according to ELN after 12 months of treatment				
C1q µg/mL	70.9 (56.1–89.7)	59.8 (48.7–67.4)	0.0141	OPT = 7, NOPT = 5
Serum cytokine levels measured at 6 months, depending on response according to ELN after 12 months of treatment				
CFB µg/mL	159.2 (90.2–194.4)	111.6 (95.1–129.5)	0.0372	OPT = 7, NOPT = 6
C5 µg/mL	12 (9.5–16.7)	9.4 (6.1–11.)	0.007	OPT = 7, NOPT = 6
Serum cytokine levels measured at 12 months, depending on response according to ELN after 12 months of treatment				
PDGF-BB pg/mL	2.6 (1.5–6)	0.82 (0.003–1.8)	0.0084	OPT = 4, NOPT = 7
CCL5 ng/mL	26.4 (6.8–63)	5.9 (0.18–22.7)	0.0281	OPT = 4, NOPT = 7
PDGF-AA pg/mL	1300 (700–1800)	450 (8.4–1100)	0.0098	OPT = 4, NOPT = 6
Angiopoietin 1 ng/mL	10.3 (6–15.1)	3.8 (0–8.3)	0.0101	OPT = 5, NOPT = 5
IFN Y pg/mL	4.9 (2.3–6.6)	1.6 (0.53–4.16)	0.0268	OPT = 4, NOPT = 6
IL-8 pg/mL	4.3 (1.952–99.7)	1.8 (0.54–5.2)	0.0404	OPT = 3, NOPT = 7
EGF pg/mL	36.2 (30.5–196.4)	10.8 (0–43.3)	0.0128	OPT = 5, NOPT = 8
VEGF pg/mL	27.8 (11.2–32.1)	12.5 (5.4–21.4)	0.0281	OPT = 5, NOPT = 8
TGF-β ng/mL	26.6 (14–52.3)	13.8 (8.6–17.5)	0.0191	OPT = 5, NOPT = 8
PDGF-AB ng/mL	4.5 (1.8–5.2)	1.2 (0.5–3.3)	0.0281	OPT = 5, NOPT = 8

**Table 5:** Levels of analytes in bone marrow samples, measured after 12 months of treatment. Median concentrations and ranges are listed. *p*-values were derived from Mann–Whitney tests.

	OPT Group (*n* = 3)	Non-OPT Group (*n* = 3)	*p*-Value
TNF-α pg/mL	9.9 (9.7–16.01)	4.7 (0.72–8.52)	0.049
IL-8 pg/mL	9.6 (6.6–77.3)	1.645 (1.2–1.7)	0.049
VEGF pg/mL	17.39 (11.9–25.8)	10.508 (4.9–10.9)	0.049
FGFb pg/mL	50.7 (46.2–116)	12.9 (10.6–26.5)	0.049
FGFA pg/mL	166 (165–239)	127 (78.1–144)	0.049
TGF-β pg/mL	24.5 (14.1–52.3)	20.1 (18.3–20.3)	0.049

**Table 6:** Significantly changed and immune-relevant serum protein concentrations between individual time points in whole cohort independently of degree of response (OPT + NOPT). Cytokine values presented as mean ± S.D. Wilcoxon signed-rank test was used to compare values obtained at different time points. Statistically significant results are bolded.

	Diagnosis (dg)	3 Months	*p*-Value dg vs. 3 Months	6 Months	*p*-Value 3 Months vs. 6 Months	12 Months	*p*-Value 6 Months vs. 12 Months
Agniopoetin-1 ng/mL	26.6 ± 19.5	16.7 ± 10.9	0.916512	13.4 ± 7.6	**0.043115**	6.2 ± 3.7	0.213525
IFNy pg/mL	3.7 ± 2.2	3.3 ± 2.8	0.600180	3.2 ± 2.7	0.144128	2.9 ± 1.9	0.310495
IL-2 pg/mL	0.11 ± 0.12	0.15 ± 0.34	0.500185	0.2 ± 0.3	0.500185	0.13 ± 0.17	0.779435
IL-4 pg/mL	2.0 ± 0.3	17.3 ± 48	0.463072	14.8 ± 42.8	0.345232	17.8 ±48.8	0.138642
IL-6 pg/mL	2.1 ± 2.2	15.2 ± 47.3	0.074736	14.8 ± 42.6	0.500185	16.6 ± 47.6	0.674424
TNF-α pg/mL	31.3 ± 20.9	11.3 ± 6.4	**0.046400**	8.3 ± 3.7	0.892738	7.0 ± 3.3	0.207579
IL-8 pg/mL	12.3 ± 25.1	16.1 ± 33.1	0.916512	12.8 ± 28.2	0.224917	11.4 ± 27.9	0.575403
VEGF pg/mL	300.0 ± 296.6	41.9 ± 31.5	**0.046400**	23.4 ± 11.0	0.138012	19.2 ± 8.5	0.109746
MMP-8 pg/mL	27,146.7 ± 46815.2	824.5 ± 953.8	**0.027709**	613.2 ± 630.2	0.892738	653.3 ± 820.6	0.085832
GM-CSF pg/mL	4.0 ± 5.8	20.5 ± 50.7	**0.027709**	16.5 ± 44.8	0.079617	14.4 ± 42.5	0.674424
Endostatin pg/mL	22,311.2 ± 11,004.2	15,888.6 ± 6521.3	0.115852	15,113.6 ± 5470.2	0.685831	16,525.1 ± 6282.5	**0.020880**
IgM µg/ml	893.9 ± 403.3	895.6 ± 589.6	0.463072	1748.3 ± 3652.0	**0.043115**	672.3 ± 295.2	0.858955
IgG1 µg/ml	3023.1 ± 976.7	3261.7 ± 1150.8	0.115852	2832.4 ± 895.6	0.500185	3004.5 ± 1170.2	0.767097
IgG2 µg/ml	2525.4 ± 706.1	2748.1 ± 577.3	0.600180	2463.3 ± 1143.9	**0.043115**	2440.2 ± 1172.6	0.593955
IgA µg/ml	1122.6 ± 575.2	1402.4 ± 401.4	0.345448	1299.1 ± 531.5	0.138012	1616.8 ± 613.3	0.858955
TGF-β pg/mL	75,524.1 ± 48,366.6	38,528.0 ± 24,429.4	0.753153	31,352.1 ± 15,667.6	0.224917	18,229.9 ± 7626.1	0.441269
C1q ng/mL	74,341.3 ± 15,950.3	66,293.3 ± 10,730.0	0.345448	57,804.0 ± 10,912.3	0.079617	62,203.2 ± 14,231.9	0.109746
C3 ng/mL	1,073,842.6 ± 33,5210.1	825,772.8 ± 319,168.6	0.248865	775,648.4 ± 233,689.3	0.685831	886,776.8 ± 52,4181.1	0.313939
C3b ng/mL	725,974.3 ± 224,693.7	709,537.7 ± 420,208.4	0.916512	527,593.8 ± 173,738.1	0.685831	570,364.7 ± 339,324.7	0.593955
C4 ng/mL	349,806.2 ± 77,609.4	290,159.9 ± 45,897.4	0.172956	258,703.2 ± 49,847.2	0.138012	297,406.6 ± 79,357.7	0.085832
FB ng/mL	161,715.6 ± 51,009.2	152,373.0 ± 25,262.8	0.345448	131,307.2 ± 29,619.4	0.224917	156,274.2 ± 46,457.8	0.138642
FH ng/mL	272,343.5 ± 43,893.3	294,454.4 ± 40,040.9	0.916512	279,224.5 ± 44,034.6	0.500185	310,911.1 ± 65,094.0	0.085832
C2 ng/mL	2135.5 ± 2767.6	505.3 ± 180.5	**0.027709**	479.9 ± 43.3	0.345232	510.6 ± 253.0	**0.017961**
C4B ng/mL	16,275.8 ± 3673.2	11,980.9 ± 4470.4	0.074736	10,681.1 ± 4371.7	0.345232	10,904.2 ± 4829.6	0.260394
C5 ng/mL	15,195.8 ± 3875.5	12,433.8 ± 2067.0	**0.046400**	10,849.2 ± 2514.1	0.685831	11,349.3 ± 3280.7	0.952765
C5A ng/mL	707.5 ± 494.5	400.3 ± 180.6	0.115852	405.9 ± 198.1	0.892738	376.2 ± 218.8	0.400815
Thrombospondin-2 pg/mL	13,770.3 ± 9169.6	9444.4 ± 5078.18	**0.027709**	14,693.4 ± 8967.9	0.079617	18,946.0 ± 10,057.7	0.020880

## Data Availability

The data presented in this study are available on request from the corresponding author. The data are not publicly available due to data privacy laws.
